# Overlay Virtualized Wireless Sensor Networks for Application in Industrial Internet of Things: A Review

**DOI:** 10.3390/s18103215

**Published:** 2018-09-23

**Authors:** Malvin Nkomo, Gerhard P. Hancke, Adnan M. Abu-Mahfouz, Saurabh Sinha, Adeiza. J. Onumanyi

**Affiliations:** 1 Department of Electrical and Electronic Engineering Science, University of Johannesburg, Auckland Park, Johannesburg 2006, South Africa; malvin.m.n@ieee.org (M.N.); ssinha@uj.ac.za (S.S.); 2 Department of Computer Science, City University of Hong Kong, Kowloon Tong, Hong Kong, China; 3 Department of Electrical, Electronic and Computer Engineering, University of Pretoria, Pretoria 0002, South Africa; A.AbuMahfouz@ieee.org (A.M.A.-M.); adeiza1@yahoo.com (A.J.O.); 4 Modelling and Digital Science, Council for Scientific and Industrial Research, Pretoria 0001, South Africa; 5 Office of the Deputy Vice-Chancellor: Research and Internationalisation, University of Johannesburg, Johannesburg 2006, South Africa

**Keywords:** Internet of Things, WSN virtualization, overlay WSN, Industrial Internet-of-Things (IIoT)

## Abstract

In recent times, Wireless Sensor Networks (WSNs) are broadly applied in the Industrial Internet of Things (IIoT) in order to enhance the productivity and efficiency of existing and prospective manufacturing industries. In particular, an area of interest that concerns the use of WSNs in IIoT is the concept of sensor network virtualization and overlay networks. Both network virtualization and overlay networks are considered contemporary because they provide the capacity to create services and applications at the edge of existing virtual networks without changing the underlying infrastructure. This capability makes both network virtualization and overlay network services highly beneficial, particularly for the dynamic needs of IIoT based applications such as in smart industry applications, smart city, and smart home applications. Consequently, the study of both WSN virtualization and overlay networks has become highly patronized in the literature, leading to the growth and maturity of the research area. In line with this growth, this paper provides a review of the development made thus far concerning virtualized sensor networks, with emphasis on the application of overlay networks in IIoT. Principally, the process of virtualization in WSN is discussed along with its importance in IIoT applications. Different challenges in WSN are also presented along with possible solutions given by the use of virtualized WSNs. Further details are also presented concerning the use of overlay networks as the next step to supporting virtualization in shared sensor networks. Our discussion closes with an exposition of the existing challenges in the use of virtualized WSN for IIoT applications. In general, because overlay networks will be contributory to the future development and advancement of smart industrial and smart city applications, this review may be considered by researchers as a reference point for those particularly interested in the study of this growing field.

## 1. Introduction

The Industrial Internet of Things (IIoT) is a recent and growing research area concerning the application of the Internet of Things (IoT) and related technologies such as Wireless Sensor Networks (WSN) in order to improve the productivity/performance of several industrial processes and systems [1]. The IIoT leverages the IoT paradigm to improve connectivity, efficiency, scalability and cost savings of different manufacturing industries and organizations. The IIoT has been recently applied in several important domains including in smart industry applications [2,3,4], smart manufacturing [5] and smart city applications [6,7,8].

Despite being contemporary, the success of the IIoT strongly depends on the advances being made in the study of WSNs [9]. This dependence exists because most IIoT deployments are mostly based on the use of sensors, which are components of the WSNs. In this regard, several existing research challenges regarding WSNs are actively under consideration in the literature. One problem is the growing heterogeneity being created by the increased development and deployment of different sensor nodes in WSNs, which prevent the co-existence of different WSN nodes on a shared physical infrastructure. This heterogeneity introduces difficulties including the interoperability problem across different administrative domains, slow deployment rates of WSNs, conflicting goals and economic interests of different WSNs node vendors, and the increasing cost of WSN deployments.

In order to address these challenges, the concept of virtualization in sensor networks has been introduced. Sensor network virtualization is aimed at providing flexibility in the deployment of WSNs, providing cost-effective solutions, improving seamless interoperability and enhancing security and management facilities. In technical terms, a virtualized wireless sensor network (VWSN) is formed by delivering logical connectivity to a subset of collaborating nodes in order to accomplish a specific task or application at a given time. These nodes are grouped based on the physical condition that these nodes may be tracking or monitoring per time. Based on the success of VWSNs, the recent concept of overlay VWSN (OVWSN) has consequently gained similar popularity, particularly for enhancing the performance of WSNs.

The concept of OVWSN, especially within the existing WSN infrastructure, will increase the reuse of different sensor infrastructures. This is possible because virtualization decouples the network infrastructure from the application services and replaces current direct connections with virtual/logical links. These logical links require the application services to choose the optimal node(s) to use in a particular situation, as opposed to the fixed allocation of nodes to a specific application task(s). The physical resources are thus allocated on-the-fly and on-demand using possible dynamic resource allocation techniques. This concept ensures that resources are efficiently utilized and shared between different application tasks.

Current and future deployment of sensor networks will require efficient and powerful devices to be used as sensor nodes. Thus, feature-rich and resource-abundant devices should be available to create a platform for VWSN, such that sensor nodes can share resources among a variety of applications to avoid unnecessary and redundant deployment of nodes. Virtualization allows users (applications and services) to use resources dedicated to them [10,11]. By complementing the concept of virtualization, overlaying further allows the creation of new services at the edge of the existing infrastructure with little or no change to the underlying hardware. A combination of virtualization and overlay services will enable robust, scalable and resilient WSNs. Besides, virtualization addresses the gap of domain-specificity in wireless sensor deployment. Thus, as WSNs become more pervasive and application areas continue to grow exponentially, the need to build OVWSNs over existing infrastructures will undoubtedly improve the implementation of resilient networks that will meet the future demands of WSN.

Thus, following the growth of OVWSN [12,13], including the increased rate of published materials in this regard, it has become necessary to provide an overview of the progress made thus far in this research area. Therefore, this paper provides a detailed review of OVWSNs, beginning with the motivation behind the need for virtualization in WSN. Then, different existing challenges are provided with an emphasis on virtualization as a possible solution. This is followed by an overview of VWSNs, its building blocks and the different types of virtualization platforms and how they comparatively perform. An exposition into OVWSN is provided based on different areas such as its topology, routing, media access and its service delivery. The state-of-the-art with regards to OVWSN is discussed. After that, few design requirements towards improving OVWSN are considered, followed by the presentation of some existing research challenges for future works. By the above, the following contributions are made in this paper:

- A comprehensive comparison of the different techniques used for implementing VWSN is provided under different categories including the various operating systems used, the middleware, and also the virtual machines used.
- The concept of OVWSN is thoroughly discussed as a potential solution to some of the problems encountered in smart industrial applications.
- Peer-to-peer topologies are well contrasted with ring topologies mainly as possible enablers for OVWSN.
- Some research challenges and potential solutions in OVWSN are also discussed for easy comprehension by budding researchers in the OVWSN study area.
- A conceptualization of the design requirements for future OVWSN has also been provided and discussed.

The rest of this paper is presented as follows: Section 2 expounds on the concepts of virtualization in WSN, and an application scenario of a smart industrial application [5] is presented. Section 3 discusses the importance of shared sensors as fundamental to the emergence of virtualized WSN. Section 4 details the core of the article on overlay virtual wireless sensor networks, while Section 5 discusses the design requirements for overlay virtual WSNs. Section 6 presents the open research areas available, and Section 7 concludes the article.

## 2. Virtualization in Wireless Sensor Networks

Technically, virtualization is set up by decoupling the application services from the underlying physical network so that the application services are not directly connected to their corresponding network elements, but are rather connected via logical/virtual linkages spread across the entire network. In most traditional schemes, the middleware and virtual machine approaches have been mostly used in WSNs to achieve virtualization at the network and node level, respectively. Virtualized WSN (VWSN) are typically applied in some recent application areas such as in smart industrial applications [14]. Due to the scale of these applications areas, for example in smart industry applications, it has become pertinent for resource sharing to be the most likely approach for deploying WSN services. Sharing of resources with less individual ownership has become the global trend [15,16], thus giving way to more resources being efficiently managed by on-demand platforms. Such shared resource management schemes have been found to be successful, for example with the Uber scheme [17], and with the hospitality scheme using Airbnb [18]. These schemes, some of which depend on sensor network infrastructures, are normally shared by a variety of users and are accessed using different platforms, for example via specialized operating systems, virtual machines, middleware and other sharing platforms [4].

**Figure 1:**
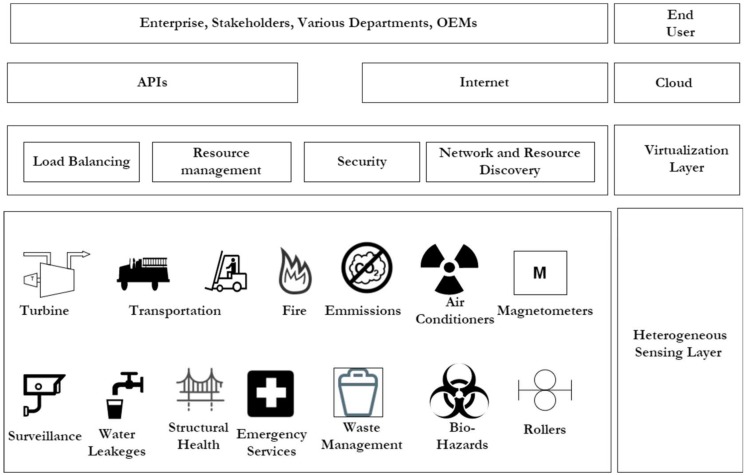
A smart IIoT concept utilizing resource sharing.

In most application areas, the ubiquity of the deployed sensor nodes places a great demand on the available shared resources. Thus, this growing demand has made the concept of virtualization of these services a highly beneficial technology for enhancing resource sharing among different stakeholders. An application area that may greatly benefit from VWSN is the smart industry application area. An example of the smart industrial concept is shown in Figure 1. It is seen from Figure 1 that by introducing a heterogeneous sensing layer, the network virtualization layer is thus able to provide fewer nodes for deployment in different sensing applications while meeting all the necessary sensing requirements.

In developing resource sharing frameworks, lightweight architectures are normally used to initiate VWSN for deployment in smart industrial applications. Essentially, these resource sharing frameworks normally include a sensing layer, a virtualization layer, cloud services and an end-user access. The sensing layer typically consists of a number of different sensing scenarios under different application domain areas [19]. The concept of VWSN in smart IIoT presented in Figure 1 aims to reduce the number of nodes required for sensing deployment. As such, the gateways are equipped with virtualization layers, which enable several application tasks to run on the gateway instead of on the sensor nodes. Thus, a great deal of load balancing is provided by the resource rich gateway for the sensor nodes.

The virtualization layer consists of resource rich gateways for the WSN. These gateways consist of a various communication standards based on IEEE 802.15.4 [20], 802.11 [21], 802.3 MAC and sub-GHz open and proprietary standards, which include 6LoWPAN [22], ZigBee [23], Bluetooth Low Energy [24], Wi-Fi, Thread [25], and ZWave [26]. The gateway is also responsible for running multiple instances of the various sensing applications on the node, while giving the illusion that only one application is accessing the gateway at a time. The gateway (or sink) is often able to provide load balancing, resource management, network discovery and able to offer a secure platform for shared infrastructure. Enterprises, governments, municipalities and civilians can thus share the data obtained from the different WSNs. Furthermore, in order to perform a particular sensing function, the number of deployed sensor nodes at the sensing layer will reduce because they are able to share a common aggregated VWSN without changing the underlying hardware network. The also supports improved scalability and reliability of the network [27].

Virtualization can be achieved at the sensor node level or at the network level [5]. Virtualization allows a sensor node to have concomitant access to several application tasks at a time [28]. Virtualization in nodes and on the network level is made possible by using operating systems, middleware and virtual machine approaches. Thus, the state-of-the-art in terms of these various approaches is provided in the next subsections.

### 2.1. Operating Systems

Operating Systems (OSs) for shared sensor networks are typically designed to alleviate the redundant deployment of sensor nodes in WSNs. We evaluate several OSs that consider resource sharing in their implementation. The design of the architecture for a WSN OS can be considered from a technical and a non-technical perspective. Technical considerations include WSN architecture, modularity, scheduling model, memory allocation, networking, programming model, debugging tools, programming language and hardware abstraction layers. Non-technical issues include documentation, certification, code maturity and licensing. A wide range of OSs for shared sensor systems is evaluated. Our review covers the following OSs: Contiki [29], RIOT [30], TinyOS [31], OpenWSN [32], LiteOS [33], nuttX [34], PAVENET [35], MANTIS [36], FreeRTOS [37], mbed OS, SenSmart [38] and SenSpire [39]. Other OSs that exist but are not covered in the table are eCOS [40], uClinux [41], ChibiOS/RT [42], CoOS [43], nanoRT [44], Nut/OS, ERIKA Enterprise, MansOS [45], NanoQPlus [46], RTEMS, Lorien [47], ThreadX [48], QNX, PikeOS [49] and Nucleus RTOS [50]. A lack of highly-deterministic performance levels are typically exhibited by the platforms provided in Table 1, which is a critical factor to be considered when developing virtual frameworks. Future platforms need to address the lack of real-time performance in OS paradigms. Tiny OS lacks dynamic resource allocation while its counterparts provide dynamic resource management.

Dynamic resource allocation is critical in decoupled ecosystems because the entire framework relies on on-demand resource utilization. Resources are not fixed to an application and thus they are shared among multiple application tasks. Virtualization techniques for resource sharing are implemented across the different OS platforms with a view to examine their efficiency levels. Contiki offers a rich networking stack that supports IoT ready protocols. It is imperative for IoT platforms to enable interactions between existing IPv4 networks, IPv6 and IEEE 802.015.4 based protocols [17]. Contiki supports µIP, which is compatible with IPv4, µIP6, IPv6, Rime stack, and with IEEE 802.15.4 related protocols. It is also imperative for the operating systems to support routing protocols for low power and lossy networks (RPL) [51] as they are integral components of the future progression of WSNs. The maintenance of sensor nodes should also be considered as it becomes daunting to maintain these nodes if they do not support over-the-air (OTA) updates. This means that for every firmware upgrade each sensor needs to be physically recalled for programming, which becomes a very difficult exercise to manage, considering the scale of future WSN. The need to support OTA is inextricably bound to the evolution of WSN towards shared ecosystems.

In addition to the specific VWSN OS compared in Table 1, we provide further discussion regarding some well-known OSs particularly considered for VWSN as follows:

(1) SenSmart: SenSmart is a sensor based OS that supports simultaneous application tasks in resource constrained nodes [38]. In order to provide concurrent execution of different application tasks, SenSmart is designed with a stack allocation system that is managed dynamically at run time. This enables an unused stack space to be reclaimed from expired tasks that no longer require it. When a new task is initiated to run, the content of the current task is compressed and saved in a circular buffer for its resumption. This mechanism typically supports the concept of virtualization in WSN as it enables more nodes to access limited system resources as required. SenSmart is an event-driven programming model and thus follows a sense-and-send workflow model. This further supports its use in VWSN. It has been implemented in some hardware platforms including Mica2/MicaZ [38]. However, it is found that SenSmart uses more CPU cycles for same applications than the TinyOS.
(2) RIOT: RIOT is an Internet of Things (IoT) specialized OS designed to support the use of diverse hardware resources in the IoT [30]. Its main aim is to provide real-time multithreading support, ensure a friendly programming model, while providing support for resource-constrained devices using low power consumption transmission technologies. RIOT is still a work in progress with no technical performance comparisons with existing Oss [30]. However, regarding VWSN, RIOT uses a realtime thread-based programming model in which different services are encoded in standard ANSI C/C++ languages to run in parallel. Thus, application tasks are encoded independently of the hardware and software in order to run them on different devices. This is a key feature required for VWSN.
(3) SenSpire: SenSpire is an event-driven and thread-based programming model [39]. SenSpire adopts a multilayer abstraction approach in order to develop networked applications. Regarding VWSN, SenSpire ensures that tasks can be programmed as events or as threads. In this case, event tasks typically have higher priority than thread tasks [39]. This ensures that the OS reacts more to external requests for system resources, thus facilitating broader use of the same system resource. It has less interrupt latency than the TinyOS, although with more overhead scheduling delay than the MANTIS OS.
(4) PAVENET: This is a thread-based OS for handling issues regarding preemption of multithreaded application tasks [35]. Its use is highly limited to the PIC18 microchip and cannot be deployed on other hardware platforms such as MICAZ. In order to support VWSN, the PAVENET OS supports thread-based programming and the use of C language. It is possible to ensure varying priority levels via the use of programmed multithreaded applications [35]. The main limitation of PAVENET is its lack of portability across diverse hardware platform.
(5) MANTIS: MANTIS is also a thread-based embedded OS that supports concurrent execution on sensor nodes [36]. It is considered for VWSN because it is completely thread-based and typically easy to program without the need to manage low-level details of the stack/memory. The time-sliced multithreading approach ensures that several application tasks can run concurrently without using a run-to-completion model [36].
(6) LiteOS: It is a Unix-like OS particularly considered for sensor nodes [33]. It adopts a hierarchical file system with a command shell that works wirelessly. LiteOS is highly flexible for VWSN because it uses a hybrid programming model that combines both simultaneous execution of application threads and events through a call-back mechanism. Application tasks can be programmed in C language [33]. Installation and the execution of application tasks is very simple and can be accomplished by dynamically copying user applications. It is highly viable for deployment in VWSN.
(7) Contiki: It is one of the most popular OSs for WSN. It provides the concept of protothreads, which combines the concepts of event-driven and thread-based approaches [29]. This allows applications and services to be dynamically uploaded/unloaded wirelessly on sensor nodes. For VWSN, Contiki is highly applicable because it supports multiple applications that are typically independent of the OS and can invariably run on top of it. Applications can be programmed in C language and updated/installed without reinstalling the entire OS.
(8) TinyOS: It is an application-specific, component-based OS that is event-driven and offers a flexible platform for innovation [31]. It is written in a variant of C-language called nesC. It may not necessarily be the most viable for VWSN because it is mainly event-driven. However, efforts are currently underway to create variants that may be suitable for VWSN.

### 2.2. Middleware and Virtual Machine-Based Approaches

**Figure 2:**
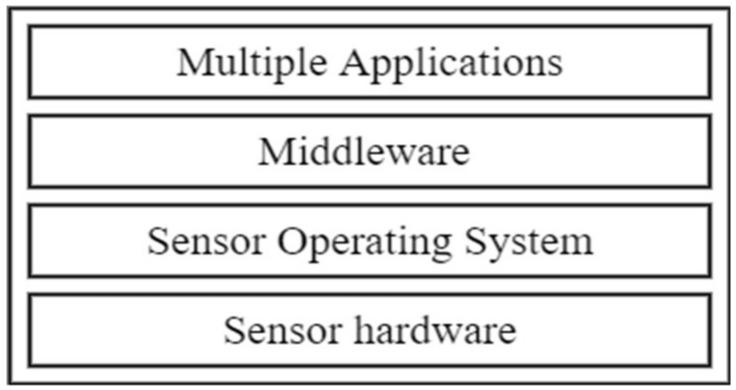
Middleware Approach.

The middleware approach affords a developer the opportunity to operate at a layer above the host OS, as exemplified in Figure 2. Middleware is intended to satisfy a wide range of system design goals that are a challenge in traditional WSN. Some of the goals are to provide a high QoS [60], ease of programming, efficient resource management [61], scalability [62], agility support, reconfiguration of nodes [63], heterogeneity [64], virtualization [65], interoperability [66], large data sets and multi-radio support [67,68]. Several academic research efforts have been put into modelling and implementing middleware for sensor nodes.

Early efforts in this regard include Impala [69], EnviroTrack [70], Mires [71], Cougar [72], Smart Message [10], MiLAN [62], TinyLime [73], DSWare [74], TinyCubus [75] and TinyDB [76]. These implementations are typically plagued by different challenges, ranging from weak abstraction support and data fusion to a lack of dynamic topologies, rigid programming models, a lack of QoS support and a limited security policy [60,77,78,79]. Some further research efforts are noted to have addressed the challenges concerning the middleware and VM-based approaches to WSNs. They yielded better efficiencies than the previous implementations. State-of-the-art implementations include among others include Agilla [80], VMStar [81], SenaaS [82], UMADE [83], Squawk VM, Mate [84], Nano-CF [44] and SenShare [85].

The programming model is predominantly threaded and event driven. Event driven paradigms enable energy conservation by only sending data/information when critical threshold conditions have been reached, otherwise the messages will not be critical and energy-worthy of transmission. The threaded model allows for the design of distributed algorithms while enabling the underlying network to conceal the heterogeneous nodes. However, programming overheads are experienced in [69]. There are limited emphases on real-time performance in the surveyed platform. Earlier implementations may not have focused on this aspect, however, both SenShare [85] and Pavenet [86] are noted to have elements of real-time performance evaluations. The dynamic nature of virtualized platforms indicates that highly-deterministic behavior is required for the success of distributed logically connected layers.

A few notable VM based solutions are briefly discussed with regards to VWSN, and summarized in Table 2, as follows:

(1) VMSTAR: It is a Java-based software framework for developing application-specific virtual machines [81]. It supports the sequential and simultaneous use of thread-based applications. For VWSN, VMSTAR does not support the simultaneous use of multi-thread application tasks, instead, it supports only single-threaded Java applications. However, concurrent events can be handled using action listeners [81]. This can be used to identify high priority threads so that expired threads can be relived of system resources to cater for other application tasks.
(2) Squawk: This is also a Java virtual machine that runs on sensor hardware [84]. Different from VMSTAR, Squawk does not require an OS in order to run, instead, all its basic requirements are inbuilt. For VWSN, Squawk adopts a different approach compared to other solutions. First, it provides an application isolation mechanism, which enables multiple application tasks to be treated as Java objects [84]. Thus, applications can have multiple threads, which are managed by the Java Virtual Machine (JVM).
(3) Agilla: It is a mobile agent-based middleware that runs over the TinyOS along with a VM engine to conduct sequential execution of multiple applications [80]. This is normally done in a round robin manner. For VWSN, Agilla depends on the TinyOS in order to provide simultaneous execution of tasks. It also guarantees this via the mobile agents executed in a round-robin style [80]. However, the difficulty in the programming language adopted by Agilla typically limits its use for VWSN. It adopts a low-level assembly-like language, which can be very difficult to modify or to build upon.
(4) UMADE: UMADE is a mechanism provided to promote fair utilization of resources among multiple contending applications [83]. It is typically built based on the Agilla VM and the TinyOS. For VWSN, UMADE typically uses Agilla for virtualization, while extending Agilla in order to provide dynamic memory management for concurrent applications.
(5) Nano-CF: It is a macro-programming framework for in-network programming and execution of multiple applications in WSN [44]. It adopts a proprietary OS called Nano-RK operating system, which enables several applications to use a common WSN architecture. This makes it suitable for VWSN. For VWSN, it allows independent application developers to write application tasks for a common WSN infrastructure [44]. These application tasks run independently and are not coupled to the sensor OS. It highly suited for data acquisition with sensor nodes having multiple on-board sensors.

### 2.3. Node Virtualization

The birth of newer sensing application areas has motivated a new ecosystem that requires the sharing of resources from a hardware perspective. The sensor node is the basic unit of a WSN and its efficient utilization is vital to the overall performance of the entire wireless network. The ability to virtualize the application services running on the network produces benefits that are analogous to those of virtualization in computing systems.

There are two strategies that have been adopted to address the virtualization of sensor nodes. These are the Sequential and the Simultaneous execution methods [87]. The Sequential execution method [87] is a rather less efficient means of virtualization because the application tasks run in sequence. In simultaneous execution, each process or task is given a time slice or quantum, and context-switching occurs based on the time slices allocated [88]. Sensor node virtualization allows for the running of several application tasks on a single node. This model overcomes the cost imperatives that accompany the redundant deployment of sensor nodes.

**Figure 3:**
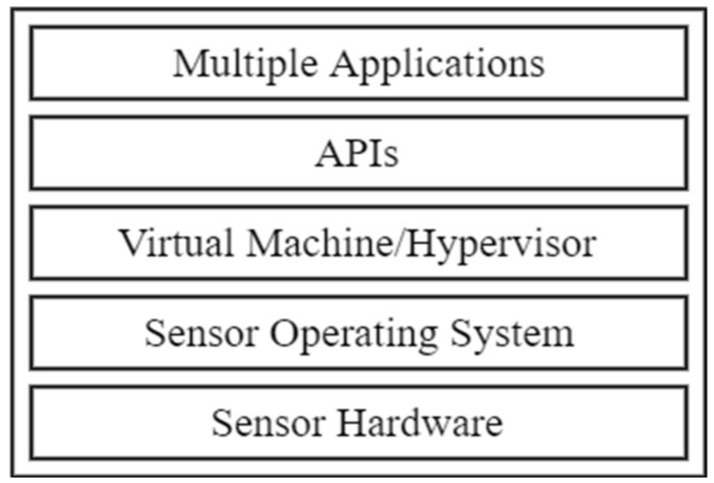
Multiple application execution on a single node [28].

Traditionally, sensor nodes are application-specific in a single domain and these sensor nodes are not used for any other application. A new sensor node is deployed in the event of a new sensing application. A major drawback associated with multiple applications sharing a sensor hardware is that the devices have limited resources. The real estate on the current sensor nodes is such that the platforms for virtualization cannot fit into the hardware resources available, particularly in terms of computation, communication and storage capabilities. Advances in processor technologies have yielded more power-efficient processors with larger memory spaces and computing power. Virtualization platforms can therefore be hosted in these nodes. Node virtualization addresses some of these challenges by creating a platform that will enable resource sharing and application management on a single node. Multiple applications can run on the node using a virtualization framework [83]. The basic concept of node virtualization is shown in Figure 3.

**Figure 4:**
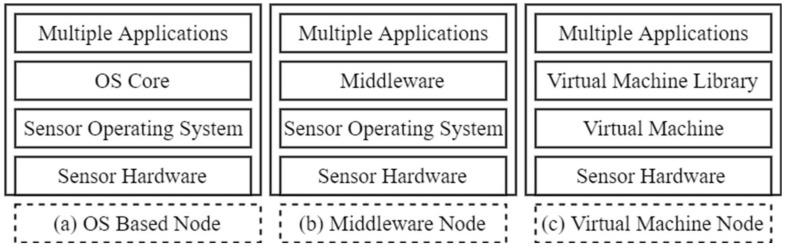
(**a**) OS-based Solution (**b**) Middleware-based solution (**c**) VM-based solution.

Virtualization can also be achieved through type 1 or type 2 virtualization. Type 1 (also called bare metal) and type 2 (also called hosted) [89] have been used in a myriad of operating systems for WSN virtualization. Node level virtualization can be achieved using sensor operating systems (OS) or virtual machine/middleware- (VM/M) based solutions [90]. These two approaches assume different architectures when realizing virtualization. In the OS approach, the OS handles the multiple services that run on the node. Event-driven and thread programming models are dominant. In the VM/M approach, the VM system operates above the host operating system. This is shown in Figure 4. The approach is split into three: OS-based, middleware and VM-based approaches.

Some efforts are noted in Table 3 for improving node virtualization based on the different OSs, the different types of middleware and the different VM-based platforms in use. Some qualitative metrics are presented to compare the different platforms. Examples of these metrics include the type of programming model being adopted by the different platforms, the consideration of resource discovery, the platform type being supported, virtualization level, heterogeneity, platform independence, multi-radio support, programming language and the communication protocols in use. Summarily, our comparison in Table 3 presents a quick view of the viability of different possible platforms available for the effective and efficient virtualization of WSNs.

The following are some of the points of interest noted from the comparison in Table 3:

(1) Event-driven programming model is more prevalently adopted for VWSN than the threaded-driven model. The is prevalence may be because VWSN nodes need to stay in the idle or sleep mode and may only be required to transmit data whenever there is a significant change in the parameter(s) being monitored. This typically makes the event-driven model more power preserving than the threaded mode. Thus, nodes can easily send signals at longer time intervals (for example, in 24 h intervals) in order to inform the network about their continuous existence.
(2) The event driven model is typically slower in execution than the threaded driven model. This makes it quite poor in managing VWSNs in highly dynamic resource environments [9]. On the other hand, a few notable threaded programming models are more capable of resource discovery, for example, the RIOT platform. For this reason, frameworks such as the RIOT platform are typically encouraged for the implementation of VWSNs.
(3) It is noted in Table 3 that some platforms lack resource discovery and publication services, which are very critical requirements in the management of the entry of new nodes into the network. Thus, platforms or frameworks, which are typically desired for VWSNs should possess dynamic management capabilities for efficient resource distribution in virtual environments.
(4) Most platforms for VWSN are typically OS based. This implies that there is a greater trend towards the use of OS-based solutions than the use of virtual-machine based solutions. This may be attributed to the higher cost of development associated with using VM than OS based solutions.
(5) Based on the examination metrics adopted in Table 3, it is quickly noted that Contiki possesses more desirable characteristics than the other platforms. It notably supports more protocols based on its unique programming model, which enables it to easily combine both the event and threaded driven models. A close competitor to the Contiki platform is the RIOT platform notable for its ability to perform resource discovery. Thus, the Contiki platform may be a more generalized model to adopt in VWSN designs.
(6) Most node level virtualization platforms in Table 3 exhibit a strong sense of heterogeneity and platform independency. For example, multi-radio support is only supported in [29,30,31], which enables various channels to be utilized leading to a reduction in the network congestion rate.

**Figure 5:**
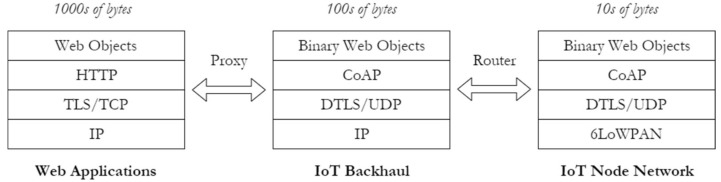
Comparison of web applications and IoT nodes interfaces.

In addition, it is worth noting that the network stack typically used in most platforms comprises of different IoT-enabled protocols that provide internet connectivity to the nodes. Essentially, these lightweight data exchange protocols for IoT paradigms can be clustered into two principal architectures, namely the broker-based [91] and the bus-based architectures [92]. In the broker-based models, the broker mediates between the publisher and subscriber. The broker is also responsible for storing, forwarding, filtering and prioritizing publishing requests. The protocols include MQTT [93], AMPQ, CoAP [94] and JMS [95]. In the bus model, the client publishes specific content to a defined subscriber without a negotiator. These classes of data exchange protocols include DDS [27], REST and XMPP [96]. Virtualized networks thrive with low payload data exchange protocols. The reduction of the payloads from thousands of bytes from web applications to tens of bytes in an IoT node results in reduced communication overheads between the nodes and the decoupled environment. This configuration leads to an improved network performance as shown in Figure 5.

### 2.4. Network Virtualization

Network virtualization allows for abstraction and sharing of WSN resources, while providing the impression of lone ownership. Network virtualization allows for the formation of dynamic logical groups of sensor nodes in which each group is set to a distinct domain. This leads to the formation of a VWSN, which is a subset of the WSN limited to a specific domain. Two approaches are discussed for achieving network-level virtualization, namely the cluster based and the overlay based approaches. The cluster-based topology achieves network virtualization by grouping logical instances of several sensor nodes from the underlying WSN infrastructure, while the Overlay networks leverages on the existing infrastructure to create a virtual topology at the application layer of the WSN. The cluster based approach is discussed in more details at this point, while the Overlay networks, being the main focus in this paper, are fully discussed in a later section.

In traditional WSNs, sensor nodes are arranged into groups called clusters. The sensors in that group communicate through the cluster head. The role of the cluster head is to communicate the aggregated information to the network sink, which in turn saves energy and bandwidth. This follows the analysis that the cost of conveying a bit of data is higher than processing it. Clustering in VWSN entails grouping logical instances of sensor nodes from the underlying network infrastructure and connecting them via virtual links to create sensor networks that are specific to an application task. The nodes are held together by logical links to form a VWSN.

Clusters are also formed by shared heterogeneous sensor nodes under different application domains. These clusters associate themselves based on their computing, communication, and processing capabilities, as well as based on their energy consumption rates. Rather than deploying private sensor systems in a particular field, these sensor nodes are shared by various end users. An example where clustering finds use is in an industrial surveillance system as part of the IIoT concept. The enterprises, stakeholders, various departments and the OEMs might need to access certain video footages. At the application layer, the relevant stakeholders can have an instance of the service they want to access. Not all end users need to deploy cameras in the respective areas, but they can share the existing infrastructure and each of them have applications running to access the surveillance system as if they are the only one accessing it.

Clustering can be realized through a static topology that offers local control and a dynamic topology that can change its network parameters on the fly. Following the clustering model, aggregated data can be sent to the sink node to reduce the number of nodes involved in the transmission. Clustering algorithms enable energy efficiency in the network and allow for improved scalability [97]. In this case, communication overheads are reduced for both single and multi-hop topologies.

## 3. Virtualization: A Solution to Some Challenges in WSN

Having presented an overview of VWSN, in this section we consider VWSN in view of some of the existing challenges in WSN. Future IIoT applications will most likely require the sharing of infrastructures as opposed to the lone ownership of the infrastructure by a specific provider. Many sectors of the global economy are shifting towards resource sharing models and the management of sharing is offered using on-demand service platforms. Traditional WSNs are deployed to a specific application area for sensing, communication, actuation, and for computational purposes. With increasing number of sensing applications, sensors might need to access data/information that lie outside the designated network or privileged data/information meant for specific users. Thus, sensor hardware infrastructures can be shared by various applications. Sharing sensor nodes are given access to the underlying hardware while being separate from each other in order to carry out their different roles. It further requires that the application tasks are not fixed to a particular node but are rather dynamically assigned to a node resource whenever they are required. Therefore, an application task is not guaranteed to use the exact sensor node it used in a previous task allocation. Access is granted using a distributed and dynamic key management technique [98] that allows multiple applications to access the underlying hardware. To this effect, the interaction between different applications and sensor nodes are not physical but logical leading to the concept of VWSN.

The existence of different applications that are logically linked to a particular hardware creates different topologies, which influences the security framework of the overall architecture. Thus, the security framework needs to be robust and resilient to enable confidentiality, availability and integrity of the application. In addition to security issues, there are other challenges that exist in WSNs and in the following subsections we present some of these challenges while considering the solutions offered by VWSN. The potential future impact of virtualization on security, scalability, quality of service, fault-tolerance, robustness and heterogeneity is discussed.

### 3.1. Security

The goal of security in any connected and communication system is to ensure authentication of the sender and receiver, confidentiality of the data, integrity of the frame and availability of the network [100]. There is a need to constantly detect and stop attacks in a WSN. Connected IoT devices enable a large number of objects to be connected directly to the internet [101]. This wide exchange of information by connected IoT devices presents an appealing environment that malicious attackers may seek to undermine. Thus, security methods are required as countermeasures. In this regard, cryptographic primitives (public and symmetric key encryption) have been employed to address security threats [102]. The role of the public-key infrastructure (PKI) provides a set of tasks, guidelines and processes. Their function is to generate, allocate, utilize and rescind digital certificates among others functions. Because Symmetric-key encryption [103] method scales at a rapid rate, it thus becomes difficult to store these keys in the sensor node. It also suffers from a poor distribution technique. Public-key cryptography [56] is very complex for implementation in computation and storage limited nodes.

A new WSN architecture was introduced in [104] that discusses authentication of certificate authorities in WSNs using a public key setup. It works by providing an initial trust key between network nodes. With ECC being WSN-ready [105], it creates a platform for authentication via virtual certificates (AVCA) as discussed in [104]. AVCA provides mechanisms to overcome issues with regards to safeguarding many dispersed networks. It fosters simplicity, interoperability, mitigation of denial of service (DoS) attacks and scalability, which is ideal for VWSN.

**Figure 6:**
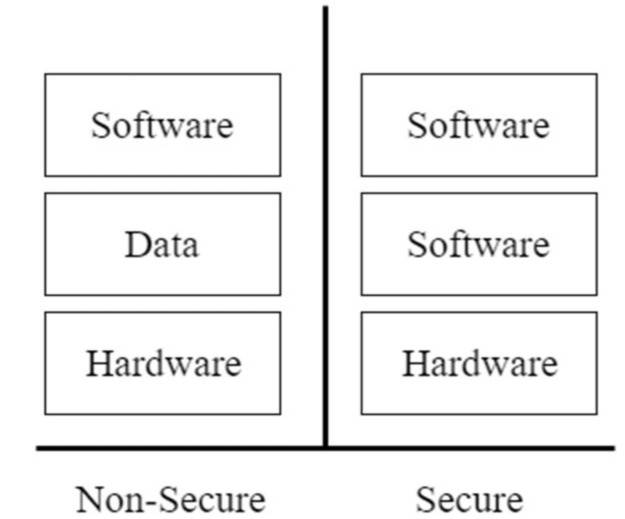
Non-secure and Secure environment.

Considering the evolution of VWSNs, the traditional internet security protocols are too resource intensive for integration into the sensor systems. Thus, lightweight security protocols are essential characteristics of any potential solution. Furthermore, security in WSN is tending towards ensuring trusted execution environments (TEE) in which security solutions are provided even from the physical (PHY) level of the OSI model [106,107]. For example, the ability for hardware partitioning in order to guarantee security suggests that virtual network frameworks can be implemented easily to avoid security attacks. Such end-to-end security approaches are enablers for VWSN. This concept is illustrated in Figure 6.

In a sensor node chipset, a non-secure world might be defined as a hardware subset consisting of memory regions, caches and specific devices. Thus, non-trusted software can be limited to an environment that prevents access to, or even knowledge of the additional hardware required to support the architecture in the secure world. TEE offers a secure and locked execution of acute applications in virtualisation environments. Furthermore, system-wide security can also be provided by integrating the trusted region into the processor that interconnects the system peripherals [108,109]. The switch between the normal and the secured worlds is performed in hardware, thus eliminating the need for a hypervisor/VMM that has processing overhead. This yields real-time performance as well as lower power operation [110].

### 3.2. Scalability

With the addition of extra hardware units to an ever expanding network, the network architecture is required to dynamically adjust its parameters to accommodate the new units without causing any performance degradation. A network is scalable if it can dynamically handle the extra load injected into the system and yet perform at an optimal throughput. It is well known that the performance rate of most networks degrades as the load increases. Thus, this could be attributed to the constricted connection between the application services and the hardware.

### 3.3. Quality of Service (QoS)

Quality of service (QoS) [111] describes the quality metrics of a wireless sensor. These metrics include the hop time of a message from one node to the other, unbalanced network traffic, network dynamics, data redundancy and energy balance, amongst others. The parameters used to configure a network typically serve to define the bounds within which the network operates in. Usually, a network’s quality is derived from the optimal performance of the network and its applications. QoS can be categorized as application-oriented QoS and network-oriented QoS [112]. Application-oriented QoS is concerned with the functions of the service tasks. The metrics of the application services include the locus of radio scope, number of active agents and the exactitude of parameters in the application. Network-specific QoS focuses on the fine grain details of the networking profile in order to ensure that minimal or no packet losses occur during the transmission or reception process. These abstractions normally inject latency and reduce reliability in the network. Virtual links in VWSN may address these challenges by mitigating the balance between the application-oriented QoS performance metrics and the network-oriented metrics. The ability to dynamically assign network components at runtime for application layer usage enables VWSN to contribute to the QoS improvement in WSN. Logical links also bring cohesion to the balance of traffic in WSN.

### 3.4. Fault-Tolerance

With the distributed nature of most WSNs, critical information bearing nodes could fail leading to an underperformance of an entire network. The failure of such nodes typically in a cluster-based topology must not affect the overall performance and operation of the network. Thus, the network architecture should have some means of self-healing and being able to recover from such setbacks. Dynamic and efficient routing algorithms should be able to reconfigure the network on runtime to accommodate the failing nodes without causing significant degradation to the network throughput. Virtual connections employed in VWSN will enable isolation of physical nodes that fail and other nodes can be assigned to the application task. A virtual approach will considerably reduce the effects of failing nodes.

### 3.5. Robustness

Resilient [113] networks are key to supporting federated sensor networks. Large-scale sensor interaction is prone to failure and these failing nodes tend to impact the network performance. A robust architecture is key to reducing these effects. The need to acclimatize to variations in the network configuration is relevant to creating robust networks. Network elements are created in modules for ease of adaptation, maintenance and isolation in the event of attacks. By considering the rate of increase in the number of application tasks in WSN, it is evident that tight coupling of services may not be the most suitable option owing to its restriction of the network from adapting to its changing sensing environment. Therefore, the decoupling of application tasks from their services and hardware and connecting them via virtual/logical links will foster a robust structure capable of adapting to the changing sensor environment.

### 3.6. Heterogeneity

The different application areas of sensor networks require that the nodes should have different physical capabilities. Some nodes have better computation and communication power [64]. Heterogeneous deployments tend to assume cluster-based [114] topologies, which require that the more powerful node should act as the leader of a certain cluster of nodes. The members of each cluster then communicate to the rest of the network via that cluster only. It is in this sense that the nodes deployed in the field for various sensing applications are heterogeneous in nature. The overall network topology and the architecture require that such provision should be made for this condition. The decoupling of sensor nodes from their respective applications implies that the virtual task manager may as well choose any node that optimally performs a certain application task without the need to use high-end nodes for simple tasks. Virtualization will in the long run promote the efficient utilization of resources.

## 4. Virtualization in WSN and the Concept of Overlay Networks

The internet was first developed as an overlay network to the traditional telephony system. It used the existing telephone infrastructure to render its services. Thus, overlays have a special history in the evolution of most technological services. Similarly, with the recent ubiquity of WSNs, it has become important to innovate over existing infrastructures in order to use these infrastructures to promote resource sharing. Essentially, Overlay networks are important because they enable transmission of data between end devices without the need to change the underlying hardware. The overlay approach is thus an appealing approach to achieving virtualization in WSN.

Olariu [115] in an early effort recognized that overlaying a virtual network over existing resources is an effective approach to deploying massive scale WSNs. Most applications and services that run on the overlay network normally execute their tasks concurrently in order to achieve resource sharing. Virtualization at the overlay layer decouples the applications and services from the existing hardware in order to give an illusion that each application task has sole control of the underlying hardware resource, while in effect it is being shared by multiple application tasks. Overlays operate at the edge of the network. They foster a distributed architecture thereby eliminating a single point of failure, which can improve network reliability and management [116].

**Figure 7:**
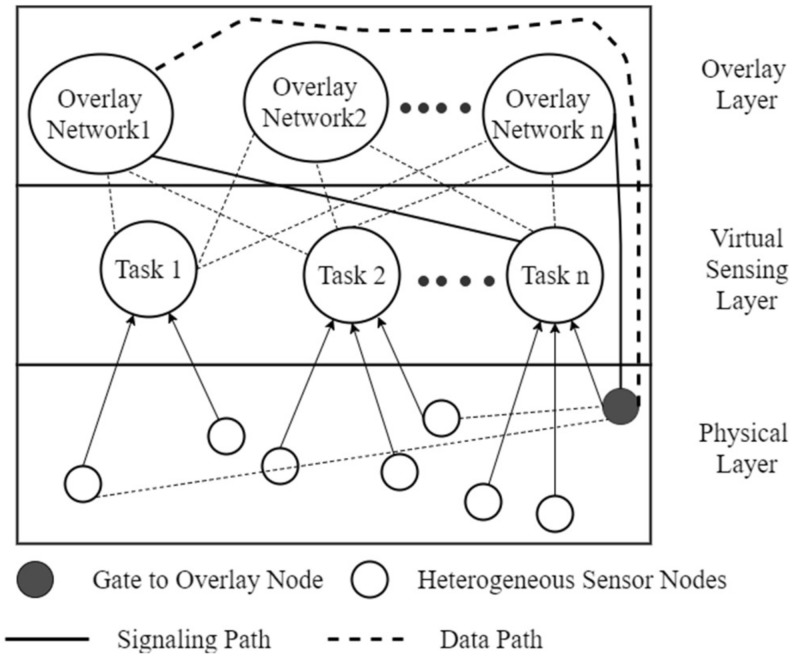
Gate to overheard virtualization technique.

Two approaches have been presented for the implementation of overlay VWSN (OVWSN), with the first been by Khan [12] in which a gate-to-overlay approach was used and the second by Xun [117] in which a gate to skip graph [118] approach was used. Khan introduced a layered design shown in Figure 7 for WSN virtualization using a gate to overlay layer. The entities in the overlay layer are grouped into logical/virtual groups that are assigned to a specific application. To address some of the shortcomings, the architecture proposed in [12] was further extended to enable interactions between VWSN Infrastructure-as-a-Service (IaaS) and Platform-as-a-Service (PaaS) for the dynamic provision of different applications and services [12]. A base ontology concept was used to build a distributed and highly-deterministic annotation of raw sensor node data in order to allow semantic applications on the VWSN. An empirical based ontology for management and creation of VWSN was discussed in [119].

An advantage of the platform is that it provides scalability in all layers. This is key for future deployments since it does not require re-deployment of the entire network in the event of new agents. The tasks in the virtual layer are loosely coupled with the agents in the overlay network, which can be expanded to multiple networks. The resource-weak nodes at the sensing layer are boosted to a higher capability using the gate-to-overlay node. There are two dedicated communication channels for signaling and data. The data channel allows for IoT-ready lightweight data exchange protocols between the interface layers. The signal path relays the network management and control related parameters, such as initialization and service management. The gate to overlay approach serves as an enabler for legacy and resource constrained nodes to participate in the virtual network area without a major infrastructural change. The drawback with this approach could possibly lie in the complexity of building the gate-to-overlay networks and the congestion and energy requirements of the node to meet the requirements of being a gateway. Dynamic formation of virtual sensing nodes strongly relies on the underlying sensing layer and their capability may be limited, hence the overload on the gate-to-overlay node.

**Figure 8:**
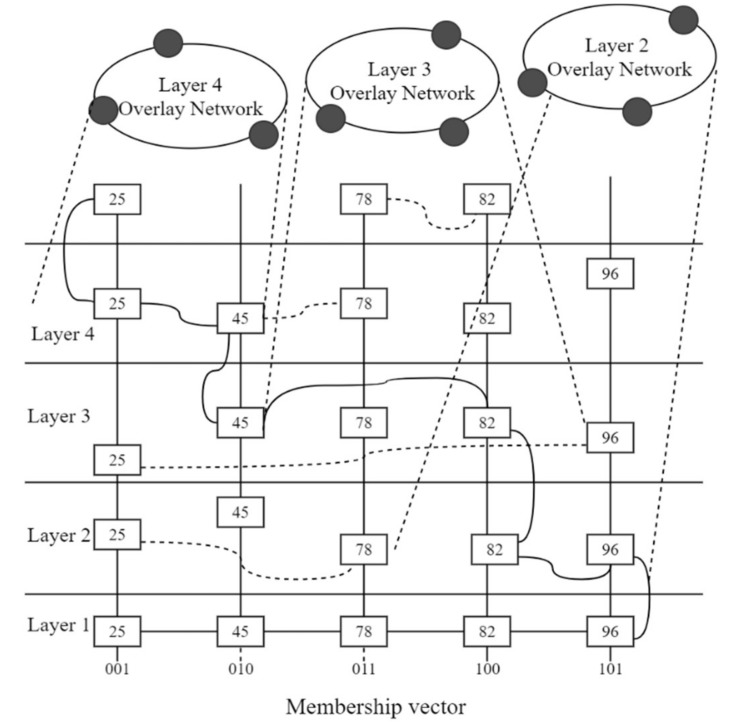
General Architecture of a Hierarchical Skip Graph Overlay Network.

Xun [117] on the other hand introduced a hierarchy–based overlay network approach leveraging on the gate-to-skip graph method as a possible approach to large scale distributed overlay networks. Earlier implementations of this approach yielded a self-aware sensing environment capable of identifying surrounding sensor nodes that join the network. This technique yielded a self-consolidating and distributed virtual sensor network. The entities of the network are used to extract the sensing resources that help to determine their fitness of participating in the overlay network. Figure 8 shows a generic approach of a hierarchy-based gate-to-skip configuration. This configuration embodies the idea that centralized control of resource management will tend to be a weakness in the system. Thus, a peer-to-peer interactive agent extension will better conceal the difference between the physical resources and the link varied in the WSN through the overlay layer. This introduces a query-like approach that sniffs the characteristics of each node in order to identify the node’s suitability to participate in the overlay network.

Nevertheless, this approach lacks a rich resource discovery capability, which affects its usefulness in large-scale federated networks. One reason for this lack may be because the resource discovery technique is range based. Thus, in the next subsections, we discuss some of the key areas in the overall architecture of overlay wireless sensor networks, which include the topology, routing, media access, service discovery, resource allocation and the utilization requirements.

### 4.1. Topology

Fei in [120] introduced the design considerations to be considered for an overlay topology in a robust overlay WSN. The goal of the topology is to for the network to remain resilient under any condition ranging from the network payload, security, communication and computation. Its design consideration addresses critical factors that influence the direction of the architecture. Shuhei [121] also noted that a peer to peer overlay topology can act as both a client and server, thereby eliminating the level of overheads associated with the large volume of information transferred between the two terminals in the network. An overlay network with a server-less architecture introduces performance degradation due to the increase in the round-trip-time of a query sent to the network. This is because it needs to retrieve a specific request as it searches every node for the required information. Multilayer overlay topologies have a potential for alleviating this burden by introducing an alternate layer of the network. The proposed topology lacks a heuristic approach for heterogeneous networks that are the characteristics of WSN. Two topologies are discussed, peer-to-peer and ring topologies.

### 4.2. Peer-to-Peer Topology

The peer-to-peer topology is more prevalent than the ring topology in deploying overlay networks in virtualized WSNs. Peer-to-peer interactions are influenced by the mutual aid of the individual nodes that form the network. This is achieved by trusted entities that do not inject malicious activities into the network. Peer-to-peer topologies also improve network performance but further introduce restricted network competences and high failure recovery times. Their implementation lacks robustness, ease of diagnosis and it has limited privacy and security [122,123].

**Figure 9:**
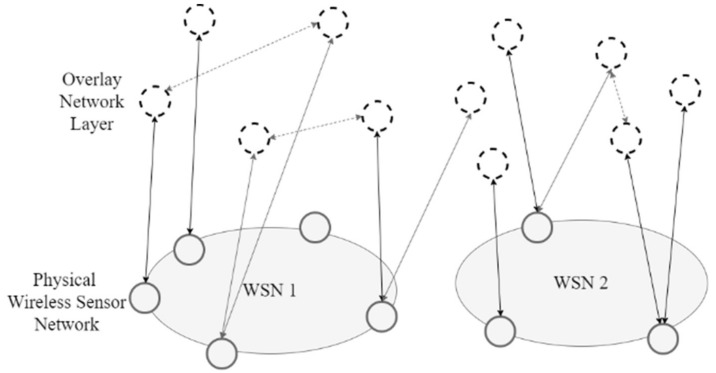
Peer-to-peer overlay topology.

Figure 9 shows a general representation of an overlaid peer-to-peer topology in a VWSN model with a distributed overlay network. The virtual nodes in the overlay have interactions with nodes from different WSNs and also between themselves.

Amy et al., in [124] proposed a structured overlay network built with in-network processing in view. The architecture considers the heterogeneity of the service providers, along with an overlay global framework that spreads across the overlay structure and stream processing for packet handling. Such architecture is critical for future networks as it offers resilient frameworks to enable resource sharing. Peer-to-peer interactions are further discussed in [125] to demonstrate the need for redundant communication paths in overlay networks to achieve resilience against nodes that can drop or be attacked via DoS [126]. The architecture of [125] further improves the availability of the network through a self-healing implementation.

Peer-to-peer topologies in overlay networks are further reinforced in [127] using a distributed model to connect end-users and gateways in mobile ad-hoc networks. Overlay networks are extended to support internet-of-things devices in a smart industrial application using a robust message exchange service for various city-wide applications such as seismic data and water leakages in the industrial distribution system [113]. The heterogeneity of the application areas in a smart industrial ecosystem renders an overlay approach very critical in addressing the scale and ubiquity of the sensor systems. It remains that the different implementation of peer-to-peer topologies presents a challenge with the vast designs that do not allowing interoperability of the different frameworks. A reference design will enable a standardized topology that can foster interoperability.

### 4.3. Ring Topology

**Figure 10:**
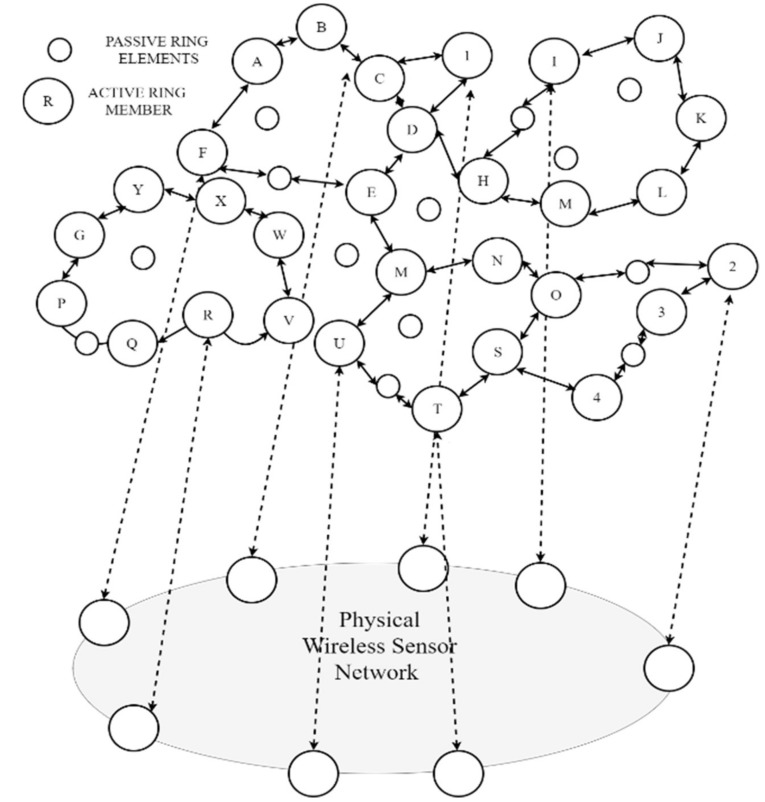
Ring Overlay Topology.

Ring topology overlay networks are presented in [128] with varying in-network architectures, which have stubs along the ring. Figure 10 shows a general ring topology in overlay WSNs. The rings consist of passive and active elements. The topology is defined by the active nodes while the passive nodes only act as a means of relaying messages between the active nodes. The passive nodes are not always used and can be bypassed. This model has nodes that are static. The advantages of a ring topology is that it can support mutual exclusion and group management [129]. The topology is advantageous because the signal strength is enhanced as messages hop from one node to the other. The nodes have equal access to the available resources and no sink node is required for central control.

The drawback of the ring topology is that it may suffer from redundant data transfers on each loop, thereby draining the energy reserves of the nodes. Furthermore, the topology is prone to single points of failures, which affects the performance of the network due to the looping effect. Figure 8 shows the active and passive elements in an overlay ring topology. The main network is shown using alphabetic characters and stubs are shown using digits.

### 4.4. Routing

Routing is a pivotal aspect of a WSN architecture. Overlay networks need to conserve energy in their choice of routing profiles. The decentralized nature and dense deployment of these virtual networks means that data needs to be transferred from the node to the sink in the shortest possible path while being energy-aware. ScatterPastry is a peer-to-peer routing protocol for overlay networks that uses distributed Hash tables [130,131]. However, it lacks discovery techniques that could possibly increase the shelf life of the network.

A greedy perimeter technique was presented in [132] that addresses the use of small chunks of data per node to relay messages in a network to enhance the shelf-life of the network. Data-driven routing algorithms typically improve the robustness of the network as depicted in [133]. The minimum connected dominating set (MCDS) is another protocol used in robust overlay networks to achieve less energy consumption rates as compared to the shortest path related protocols [134].

### 4.5. Media Access

Spectrum access techniques in overlay networks are typically distinguished between the time frequency and the time division channels approach [135]. Likewise, channel access for overlay networks can be achieved via the use of intelligent techniques. As VWSNs continue to scale, the communication channel access will thrive using intelligent mechanisms for the management of access channels used in the overlay services. Chiti et al., in [136] proposed a versatile channel selector for overlay networks. Channel selection methods in traditional WSNs typically include time slotted channel hopping [137]. The use of cognitive radio methods uses an energy detector to select the communication channel [116]. Similarly, Game theory techniques also use channel selection methods [138,139]. This reduces the probability of channel access collisions by overlay applications and services. However, the game theory method may become inefficient as the network scales up.

### 4.6. Service Discovery

Services in WSN could be thought of as any object that sources information or manages a resource on behalf of another. Service discovery in overlaid virtual WSN could be centralized or decentralized [140,141,142] owing to the network architecture and by the purpose of the network. A service centric model for overlay VWSNs is proposed in [142] and contrasted with a network centric model prevalent in traditional WSN service discovery techniques. The service centric model was shown to yield improved congestion and better energy management as compared to the network centric model. Broadcast mechanisms are also discussed in [143,144,145], application layer protocols are described in [146,147], while reinforcement learning is presented in [148] in order to design robust frameworks for the OVWSN.

### 4.7. Resource Allocation and Utilization

Carmen et al., in [149] discussed about a framework for resource allocation in a virtual sensor network. As the number of applications increased, it was shown that resource allocation complexity emerges. This was driven by the fact that there is limited real-estate in the sensor node for computation, communication and energy management. The need to re-use the existing infrastructure has given rise to novel paradigms that address resource allocation and utilization. Sharief et al., in [150] also discussed about a three tier approach to efficient utilization. However, the implementation in [150] is collocated near the edge resource of the network, which poses a challenge for its use in shared sensor networks.

### 4.8. Summary

The overlay approach to VWSNs may still be considered as being recent, and for now are largely driven by peer-to-peer interactions. These interactions foster a server-less approach to data exchange between nodes. Resource limited nodes are thus capable of participating in virtual overlay networks by adding a gate-to-overlay node to enable buffering of the resource-limited node by the capable node. It is further noted that the hierarchy based skip graph overlay may be a favorable alternative to the modeling of the overlay network. It was also noted that the peer-to-peer topology for overlay design is more widely adopted than the ring and other topologies. This is because they are considered to be trusted counterparts that do not inject malicious activities into the network. Further notes include the fact that routing protocols for overlay networks may need to address the dynamic and loose couplings associated with overlay networks, while ScatterPastry and the distributed harsh table are quite applicable in peer-to-peer interactions. With the physical disconnect between the overlay layer and the underlying infrastructure, service discoveries have to be dynamic in order to manage the assets in the ecosystem. Finally, it was noted that the resource assignment in the VWSN is a volatile process and may thus be assigned based on-demand.

## 5. Overlay Virtualized WSN: Some Design Requirements

The use of Overlay networks in shared sensor networks will require the deployment of resource rich hardware. According to Moore [151], the processors are getting more efficient and faster. Thus, sensor nodes may soon be less limited by the available resources at the hardware level. Advances in silicon fabrication will also enable sensor nodes to be resource rich and energy efficient. Following this, it is envisaged that elastic network designs will ensure that routing protocols are capable of catering for the demands of virtual network implementations. As more multiple applications concurrently access the available resources, there is need to have a robust resource management and utilization framework. This framework will be able to adjust to the changing demands of the network. The co-existence of resource rich and constrained nodes will entail a high level of heterogeneity support.

Khan et al., in [12] addressed the challenge of heterogeneity support by adding a gate-to-overlay node. This node assists other resource limited nodes to participate in the overlay network via cooperation. Such an implementation introduces complexities in the architecture. The application services need to be isolated to ensure that the entities of the next application does not intrude into the next application without relevant authorization. This is done in conjunction with the security framework that needs to be put in place to mitigate the risks of concurrent execution of applications. Nodes that enter the network at runtime will be managed through the same security framework.

As the network scales on the physical side, the overlay architecture should be able to adjust dynamically to the underlying hardware. Connections between the overlay network and the physical network are not permanent are normally established on-demand. Consequently, their reliability becomes a function of the QoS.

Over-the-air (OTA) firmware updates remain a crucial aspect of the design requirement, as they enable wireless reprogramming. Generally, sensors should not be recalled from the field in order to conduct a firmware upgrade. Thus, a modular design will provide an ease of programming OTA. Modularity will enhance the system design and provide pluggable units that make maintenance and development manageable and efficient.

Furthermore, network interfaces on the application side should be IIoT compliant [152] by being ready and lightweight with a robust interoperability context. In this regard, resource discovery plays a pivotal role in the overall architecture of an OVWSN. Concurrent resource requests from different applications demand that the discovery techniques be highly efficient and flexible. The creation of the overlay topology should be fault tolerant and immune to single-point-of-failure problems. OpenThread is a potential solution to the self-healing demands of network topologies offering a mesh topology.

Following the above notes on some design requirements for OVWSN, the following summary is provided:

(1) *Multiple application execution*—The loose coupling between the application tasks in the overlay network and the underlying infrastructure enables dynamic resource sharing and for several applications to utilize same hardware with the illusion of lone ownership, which forms the core of shared sensor systems.
(2) *Robust and IoT ready routing protocols*—Several lightweight routing protocols exist in the IoT domain that are poised to take the lead in framing the standard protocols for data exchange in WSN. A few protocols like CoAP [153] have exhibited dynamic response to their operation in virtual frameworks as well as in overlay networks.
(3) *Resource-rich nodes*—Recent advances in silicon technologies have produced a rise in the number resource rich sensor nodes being used for WSN. With the increase in the demand for shared sensor systems, it is imperative that resource rich nodes allow for energy efficiency, yet producing high throughput. Sensing applications such as in video, seismic, terrestrial and volcanic activities suggest that high- end devices are needed to process these application areas. Consequently, multi-core embedded systems [151] are noted to have contributed to the progress in resource-rich physical layers. The aggregation of data before it is sent to the sink node is also required for these nodes to avoid redundant data being sent to the node. Only significant data/information is sent, which could signify a change in status from the previous value.

## 6. Overlay Virtualized WSN: Open Research Challenges

The ubiquity of sensor networks has given rise to the need to build overlay services and virtualize sensor nodes to reduce redundant deployment of nodes. With increase in the demand for more resources, there is urgent need to shrink the cost of deploying sensor nodes and to share the available hardware resources that these networks utilize. The starting point to limiting the increasing cost of hardware deployment is to improve sensor node sharing. The scale of sensor node deployment has led to the need to reuse the deployed hardware for new application areas. With virtualization allowing for the sharing of resources, applications, services and infrastructure, several problems and challenges arise. A few of these challenges in overlay WSN are presented as follows:

### 6.1. Real-Time Performance

The introduction of virtualization framework into the node’s OS creates overheads that limit the performance of the OS. It is observed in Table 3 that PAVENET and SenShare are proponents involved in providing real-time performance in their technologies. Other models were noted to lack such capabilities. Thus, it is imperative to move towards near-real-time performance.

### 6.2. Advanced Node Virtualization

With the boom in the need to share sensor resources and build overlay networks on top of them, opportunities exist for deeper virtualization of discrete sensor layers like the MAC layer and routing layers. The efforts in [154] has proposed a service provisioning platform for cutting-edge middleware design that is aimed at a scalable, sustainable and secure virtualization platform, but no further work has been undertaken to realize this concept.

### 6.3. Publication and Discovery

Virtualized environments normally experience the complexities associated with implementing service discovery and publication. Virtual environments are available on demand and are terminated when they are no longer needed. This creates a need for dynamic management of resources used in the virtual environment. However, in conventional WSNs, a resource location and discovery service [155] coupled with CoAP provides for the standard peer-to-peer connection that does not rely on federated services for resources discovery. An implementation of this nature will address the challenges faced with publication and discovery in virtualized nodes.

### 6.4. Simulation Tools

The large deployment scale of sensor networks makes it difficult for new protocols and algorithms to be tested in typical physical network implementations. Simulation environments are thus needed to test and optimize the proposed algorithms and protocols. Simulators such as COOJA [156], XMOS and AVRORA [157] offer reliable and acceptable performance for WSN preliminary design. Efforts have been conceived to address aspects of simulators in virtual sensor systems in [158,159]. However, these are discrete approaches addressing aspects of the virtual environment. An integrated simulation framework offering end-to-end services is presented in [160]. This is platform-independent and comprises of a variety of different network APIs to provide a common communication paradigm. It also has the ability to split application requirements into events, time, query and data sections. It remains pertinent to create scalable and robust platforms to cater for the on-demand behaviour of virtual sensors.

### 6.5. Task and Sensor Node Assignment

A correlation exists between the types of sensors to be selected to perform a certain task in a WSN. This is based on the current task to be performed and the future sensing applications in which the node might be involved. A sensor-mission assignment framework is proposed for wireless sensor systems that use energy-conscious allocation algorithms [161]. The need to extend this allocation scheme to multiple applications that share a single node will enable shared sensor systems to efficiently and optimally assign nodes to their tasks.

### 6.6. Evolution of the Framework

Dynamic and evolutionary platforms are required for future virtual nodes. In recent years, WSNs and related technologies have been evolving towards the use of IoT [162]. Sensor nodes need to further evolve towards the use of overlay virtual nodes. Future sensor nodes need a framework that can adjust to new sensing environments without being reprogrammed. This could possibly be introduced through the use of artificial intelligent ecosystems in sensor nodes [163]. Future sensor node virtualization networks are expected to embrace machine learning [164] and deep learning [165] techniques in order to adapt to different sensing environments without the need for any hardware upgrade.

### 6.7. Abstraction Support

Heterogeneous implementations are typical in most WSNs. The different nature of hardware systems presents a challenge to the architecture of the operating system and its related packages. Different hardware vendors require different software routines in order to emulate the underlying hardware. This introduces complexity into the software design. A unified abstraction layer supporting heterogeneous platforms will reduce the complexity of the architecture and number of subroutines.

### 6.8. Energy Efficiency

Ensuring that sensor nodes are energy efficient is essential to the success of WSN deployments. While it is evident that future nodes will have more resourceful processors, energy-aware communication protocols and routing algorithms are necessary for the longevity of nodes in the field. Radio duty-cycling has been in the forefront of most energy conservation techniques, but it might not be the optimal approach to large scale overlay virtual networks. Thus, better energy efficient techniques and algorithms are required for OVWSNs. In addition, it is envisaged that energy efficient methods for resource management in OVWSN will be a point of concern in future designs. One notable effort in this regard can be found in [166] in which authors proposed a joint failure recovery, fault prevention and energy efficient resource management architecture for software defined networks (SDNs). In this regard, the energy consumption and reliability of selected paths are optimized, while maintaining the required Quality of Service (QoS) of the network. This work typically highlights some possible future research solutions that can be considered towards the realization of a more energy efficient OVWSN. Another recent work worth noting can be found in [167] in which authors considered the case of joint energy efficiency, QoS-aware path allocation and Virtual Network Function (VNF) placement for SDN. In this regard, authors propose a novel resource (re)allocation architecture to improve energy efficiency in SDN based networks. It is shown that the proposed solution achieves near optimal solutions particularly in terms of the execution time for real-life network deployment. Such effective solutions as in [167] will find future importance and application in OVWSN.

### 6.9. Security, Resource Management and Allocation

Security, resource management, as well as computational latency remain areas of concern in the quest to virtualize sensor nodes. The OSs, VMs and the middleware built for sensor node virtualization face the challenge of assigning and arraying applications in shared networks. Resource management seeks to enable efficient utilization of the underlying hardware that gathers data from the environment. The ubiquity of future sensor nodes and their connectivity to the internet will introduce a security and privacy component that needs to be aligned to the existing and future internet security layers. Lightweight security schemes need to be adopted that will provide the same integrity, confidentiality and availability as provided by the mainstream internet security framework.

Virtualized platforms at the sensor node will enable efficient resource utilization and conservation of the number of deployed nodes. Thus, dynamic frameworks are relevant for adaptive WSNs, which are networks that will be able to handle many applications on a single node. This will require that open communication standards are embedded in the network stack. Dynamic allocation and management of resources will be crucial in these future implementations. Efficient resource management and scheduling protocols for resource reservation and session management will ensure that real-time performance is guaranteed on the virtualized platforms.

### 6.10. Process Scheduling

Efficient process scheduling algorithms for sensing activities are also vital to minimizing energy losses in OVWSNs and are therefore important in the virtualization of sensor nodes. Large-scale federated sensor network platforms are potential considerations that will usher in the virtualization of sensor nodes. Connectivity between heterogeneous virtual sensor nodes will play a pivotal role in this paradigm and this is an area for further investigation.

### 6.11. Challenges from Other Emerging Technologies

There are several other emerging technologies designed with the aim to enhance the IIoT. An example of such a technology is Fog Computing (FC). In particular, FC has been recently applied in WSN with the goal to improve the performance of the IIoT. An extensive review was presented in [160] with regards to the merger between FC and the IoT. One challenge noted in [160] relates to the handling of the unpredictable large volume of data generated by different IoT based applications. It was noted that proximate Fog and remote Cloud data centres may present possible solutions to this problem. These are concepts much related to the development and use of VWSN. An energy efficient algorithm for fog-supported WSN was proposed in [161]. It was shown that the algorithm improved the network lifetime of WSNs by 40% compared to other known algorithms. Such algorithms can be further developed for inclusion in the OVWSN concept with the aim to improve the IIoT. A Fog supported smart city network architecture termed FOCAN was proposed for management of applications in the IoT environment in [162]. The FOCAN architecture presents as an interesting framework for application in OVWSNs. It is envisaged to further address the high energy consumption rates being experienced in the use of WSN in different IIoT applications, particularly those involving the use of the OVWSN. These new emerging technologies have a promising future in the application of OVWSN in IIoT. Finally, as Industrial IoT starts to included new emerging concepts, such as networks for multimedia and big data [168,169], OVWSN will also need to adapt to allow for QoS and high volume traffic.

## 7. Conclusions

It is envisaged that the use of sensor systems in the industrial internet of things will drive the next level of industrial productivity. These sensors will initiate tasks and communicate with other equipment in order to lower operational costs, prevent accidents and failures during operation and potentially take action in dangerous scenarios. One key technology that will significantly contribute to this vision is the overlay virtualized wireless sensor networks (OVWSN). The deployment of OVWSN will advance the use of shared sensor system towards reducing the redundant deployment of sensor nodes for different sensing applications. Overlay networks are vital to the success of the IIoT as they will provide a platform for the reuse of existing infrastructures in order to offer robust and dynamic services. Given the ever-growing study of OVWSN, we have in this paper presented a review of VWSN and the use of overlay networks concerning their application in IIoT. The various forms of shared sensor techniques were discussed. The concept of overlay services at the edge of the existing virtual wireless sensor network was also reviewed. It is noted that the efficient utilization of WSN resources is vital and the efficient management of resources will guarantee the real-time performance of the virtualized platforms. In general, because overlay networks will be instrumental in the future development and advancement of smart industrial and smart city applications, this review may be considered by researchers as a reference point for those particularly interested in the study of this growing field.

## Figures and Tables

**Table 1:** A comparison of some of the operating systems of interest.

Platform	Contiki	TinyOS	MANTIS	OpenWSN	LiteOS
**Real-Time**	No	No	No	No	No
**Hardware Platforms**	ESB, TelosB, Tmote Sky	MICA (z)(2), TelosB, Iris, Shimmer	MICA(2)(z), Telos, MANTIS nymph	TelosB, GINA, WSN430, Z1, OpenMoteCC2538	MICAz, IRIS
**Virtualization**	Serial Execution	Yes	Semaphores	Yes	Synchronization primitives
**Static or Dynamic**	Dynamic	Static	Dynamic	Dynamic	Dynamic
**Network Support**	uIP, uIP6, Rime	Active message	Comm	6LoWPAN, RPL, CoAP	Message-based
**Simulation**	Cooja. MSP-Sim, NetSim	TOSSIM, Viptos, Qualnet	XMOS	Open Visualizer, OpenSim	AVRORA
**OTA**	Yes	Yes	No	Yes	Yes
**Latest build**	2.2.1	2.0	1.0 Beta	1.8.02	1.0
**Multi-threads**	Yes	Tiny-threads	Yes	Yes	Yes
**Release date**	2004	2000	2005	2011	2008
**Concurrent execution**	Yes	Yes	Yes	Yes	No
**References**	[52,53,54]	[31,55,56]	[36,57,58]	[32,59]	[37]

**Table 2:** Comparison table for wireless sensor middleware and VM-based platform.

Platform	SenShare	Pavenet	Agilla	Squawk VM	VMStar
Programming Model	Event-driven	Thread	Tuple-space and mobile agents	Thread	Thread
Real-time Performance	Yes	Yes	No	No	No
Communication Protocols	CTP	Not discussed	Not discussed	6LoWPAN, CTP, LQRP	Not discussed
Decoupling	Yes	No	Yes	No	No
Programming Language	nesC	C	Assembly	J2ME	Java

**Table 3:** A comparison of different virtualization platforms.

Platform	Programming Model	Resource Discovery	Type	Heterogeneity	Platform Independence	Multi-radio Support	Programming Language	Protocols
**Contiki** [99]	Protothreads	No	OS	Yes	Yes	Yes	C	HTTP, COAP, UDP, TCP, RPL, 6LoWPAN
**RIOT** [30]	Threaded	Yes	OS	Yes	Yes	Yes	ANSI C/C++	6LoWPAN, RPL
**TinyOS** [31]	Event-driven	No	OS	Yes	Yes	Yes	nesC	6LoWPAN, ZigBee
**OpenWSN** [32]	State-machine	No	OS	Yes	No	No	C	6LoWPAN, RPL, CoAP
**FreeRTOS** [37]	Threaded	No	OS	Yes	Yes	No	C	Third-party network stacks
**VMStar** [81]	Threaded	No	VM	No	No	No	Java	NA
**SenaaS** [82]	Event-driven	No	VM	Yes	Yes	No	NA	NA
**SenSmart** [38]	Event-driven	No	OS	Yes	Yes	No	nesC	NA
**SenSpire** [39]	Event-driven and threaded	No	OS	Yes	Yes	No	CSpire	CSMA, CSMA/CA, B-MAC, X-MAC
**Agilla** [80]	Tuple space and mobile agent	Yes	VM	Yes	No	No	Assembly-like	NA
**LiteOS** [33]	Event-driven and Threaded	No	OS	Yes	Yes	No	C	NA
**PAVENET** [86]	Threaded	No	OS	No	No	No	C	NA
**MANTIS** [36]	Threaded	No	OS	No	No	No	C	TDMA
**UMADE** [83]	Event-driven	No	VM	No	No	No	nesC	NA
**Squawk VM** [83]	Threaded	No	VM	No	Yes	No	J2ME	CTP, 6LoWPAN, AODV, LQRP
**Nano-CF** [44]	Event-driven	No	VM	Yes	Yes	No	Nano-CL	DSR, TDMA, B-MAC
